# The biological roles and molecular mechanisms of m6A reader IGF2BP1 in the hallmarks of cancer

**DOI:** 10.1016/j.gendis.2025.101567

**Published:** 2025-02-20

**Authors:** Li Qiu, Shourong Wu, Lei Zhang, Wenfang Li, Debing Xiang, Vivi Kasim

**Affiliations:** a Key Laboratory of Biorheological Science and Technology, Ministry of Education, College of Bioengineering, Chongqing University, Chongqing 400045, China; b Department of Oncology, Chongqing University Jiangjin Hospital, Chongqing University, Chongqing 402260, China; c School of Pharmaceutical Sciences and Institute of Materia Medica, Xinjiang University, Urumqi, Xinjiang 830017, China

**Keywords:** Cancer hallmarks, IGF2BP1, m6A modification reader, Molecular therapy

## Abstract

N6-methyladenosine (m6A) is the most abundant and well-investigated internal RNA modification in eukaryotic RNAs, affecting its target gene expression by controlling RNA localization, splicing, stability, and translation. m6A modifications are regulated by m6A methyltransferase complex, demethylase, and reading proteins. Insulin-like growth factor-2 mRNA-binding protein 1 (IGF2BP1), a member of a conserved family of single-stranded RNA-binding proteins, has recently been identified as a vital m6A reading protein. IGF2BP1 is highly expressed in various tumors and is associated with poor prognosis and treatment resistance. Furthermore, previous studies have shown that IGF2BP1 plays critical roles in regulating various cancer hallmarks, including sustained cell proliferation, cell death resistance, activation of invasion and metastasis, deregulated cellular energetics, immune evasion, and unlocking phenotypic plasticity. IGF2BP1 could promote the expression of cancer-related genes by recognizing their m6A sites, thereby altering cell characteristics, and eventually, malignancy. Therefore, IGF2BP1 might be a potential target for tumor diagnosis and anti-tumor therapeutic strategies. This review summarizes the current knowledge on the functional roles and underlying molecular mechanisms of IGF2BP1 in regulating cancer hallmarks. Moreover, we discuss the prospects of IGF2BP1 as a potential tumor diagnosis marker, as well as a potential target for an anti-tumor therapeutic strategy.

## Introduction

Epigenetic modifications such as chromatin remodeling, histone modifications, RNA methylation, and DNA methylation play critical roles in regulating gene expression and thus are involved in numerous biological pathways, including reproduction, differentiation, development, and senescence.^1^, ^2^, ^3^ Furthermore, impaired epigenetic modifications are also closely related to various diseases and pathological conditions, such as post-stroke depression, autoimmune diseases, neurological diseases, and cancers.^4^, ^5^, ^6^, ^7^, ^8^, ^9^ RNA post-transcriptional modifications are a series of chemical modifications of RNA molecules after their synthesis. These RNA modifications have significant influences on the stability, localization, splicing, translation, and degradation of RNA, and thus are one of the pivotal mechanisms for regulating intracellular gene expression.^10^^,^^11^ Among them, methylations at the N1 position of adenosine (m1A), N5 position of cytosine (m5C), and N6 position of adenosine (m6A) have received increasing attention these years.^12^^,^^13^ The functions of RNA methylation depend on the type and location of the RNA in which it occurs, with regulations on RNA stability, RNA splicing, and RNA translation as its main functions. RNA methylation is involved in many physiological functions such as cell immunity, differentiation, development, and senescence, and is closely related to various pathological conditions.^14^, ^15^, ^16^, ^17^

**Figure 1:**
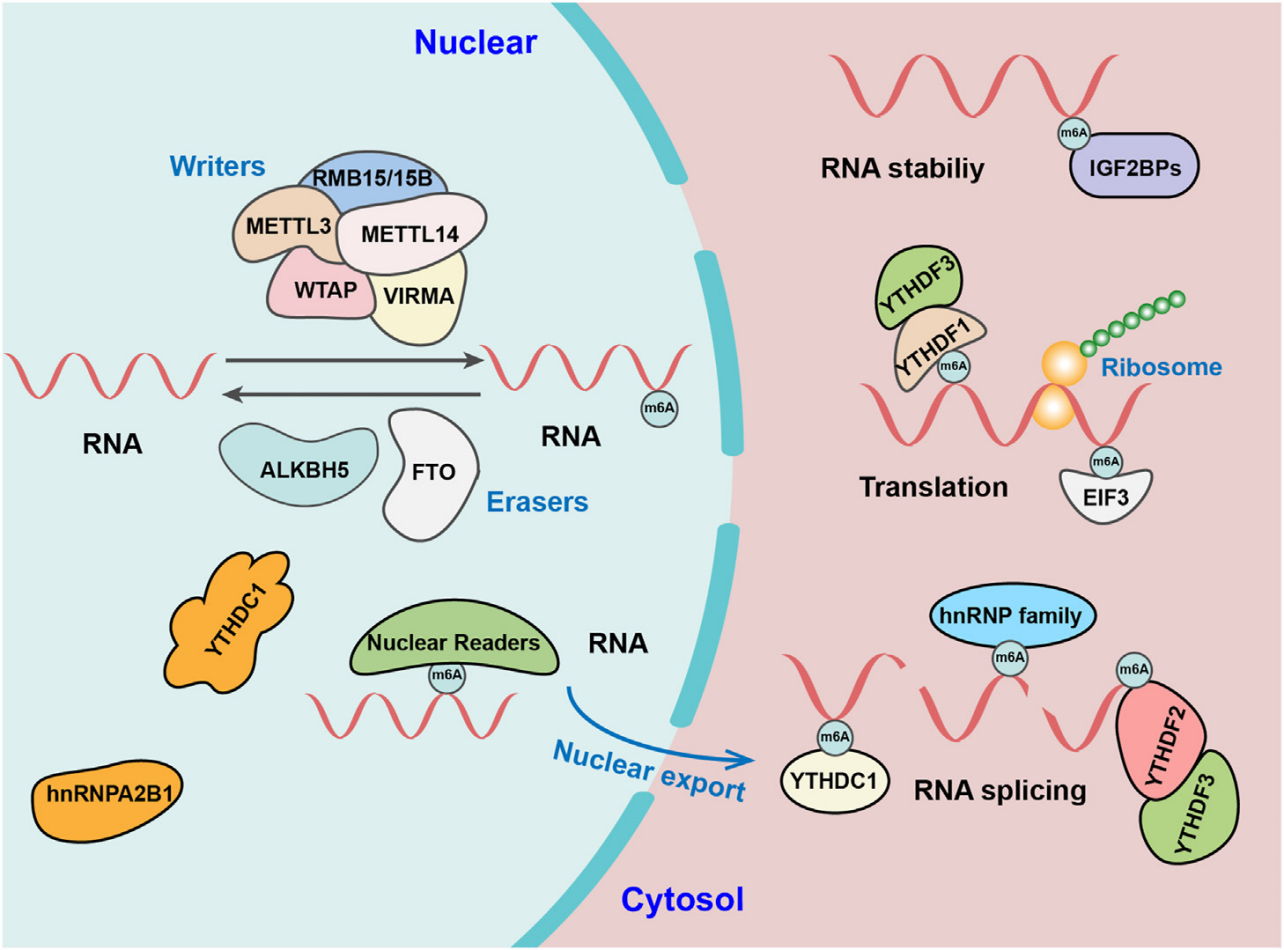
Working model of m6A regulator (writers, erasers, and readers) on m6A modification.

m6A modification is the most abundant conserved internal modification in eukaryotes which affects nearly the entire lifecycle of RNAs, including RNA export, RNA alternative splicing, RNA stability and degradation, RNA localization, as well as translation efficiency.^18^^,^^19^ m6A frequently presents in the consensus motif RR (m6A) CH, in which R is guanine or adenine and H is adenine, cytosine, or uracyl, and mainly clusters around stop codons and 3′ untranslated regions (3′-UTRs).^20^^,^^21^ m6A modification is a reversible regulation process, which is regulated by m6A methyltransferases (m6A writers), m6A de-methyltransferases (m6A erasers), and m6A reading proteins (m6A readers) (Fig. 1). The m6A writers, including methyltransferase-like 3 (METTL3), methyltransferase-like 14 (METTL14), Wilms tumor 1-associated protein (WTAP), zinc finger CCCH domain-containing protein 13 (ZC3H13), vir like m6A methyltransferase associated gene (VIRMA), and RNA binding motif protein 15/15B (RBM15/15B) can catalyze RNA methylation; while the m6A erasers, such as Fat and obesity-related protein (FTO) and alkB homolog 5 (ALKBH5) remove the m6A methylation. Subsequently, m6A sites are specifically recognized by the m6A readers, such as YT521-B homology (YTH) domain family 1–3 (YTHDF1–3), YTH domain containing 1–2 (YTHDC1–2), insulin-like growth factor 2 mRNA-binding proteins (IGF2BPs), heterogeneous nuclear ribonucleoprotein family, and eukaryotic translation initiation factor 3 (eIF3), which belong to a class of RNA-binding proteins that can specifically bind to m6A-methylated mRNAs. This will eventually regulate target mRNA expression and trigger a series of downstream biological processes.^22^, ^23^, ^24^, ^25^ The reversible regulation of m6A has been found to play an extremely vital role in different human diseases including obesity,^26^ heart failure,^27^ Alzheimer's disease,^28^ heart disease,^29^ and nonalcoholic fatty liver disease,^30^ as well as human cancers including lung adenocarcinoma, esophageal squamous cell carcinoma, and hepatocellular carcinoma (HCC).^31^^,^^32^

**Figure 2:**
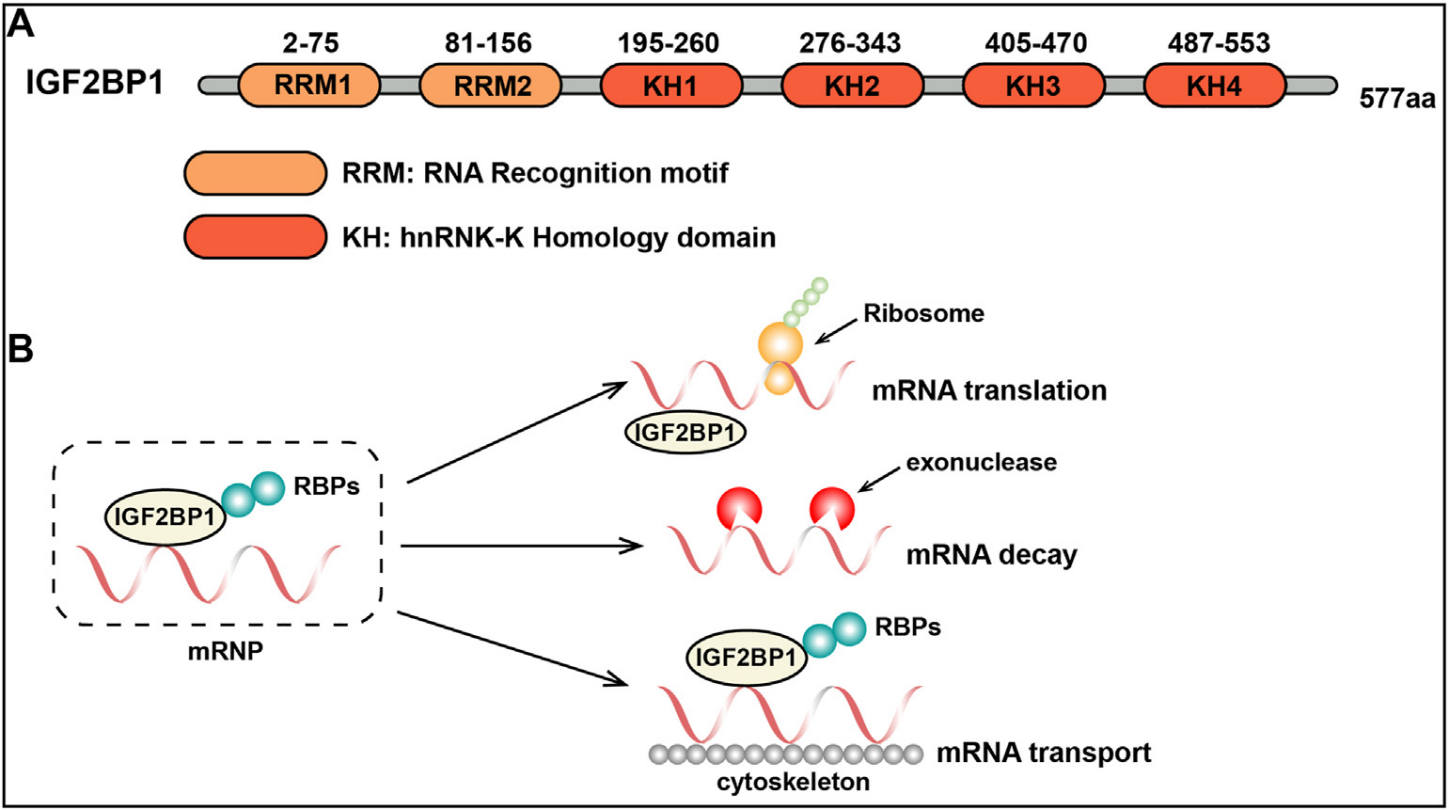
Regulation of IGF2BP1 on mRNA stability and mRNA translation. **(A)** Domain structure of IGF2BP1. IGF2BP1 contains two RRM domains (orange) and four KH domains (red). **(B)** IGF2BP1 binds to target mRNAs and RNA-binding proteins (RBPs) in the cytoplasm to regulate mRNA transport, stability, and translation by recruiting transcripts into protective messenger ribonucleoprotein particles (mRNPs).

The m6A reader protein IGF2BP1 (insulin-like growth factor 2 mRNA binding protein 1) is initially identified as a 75 kDa polysome-associated protein that can promote c-MYC mRNA stability through binding to its coding region determinant.^33^ IGF2BP1 prefers to recognize m6A modification in the “RRACH” motif, where R, A, C, and H represent purine, methylated adenine, cytosine, and non-guanine bases, respectively. However, compared with other m6A readers such as YTHDFs, YTHDC1, and YTHDC2, which have more stringent sequence requirements, the sequence specificity of IGF2BP1 is not absolutely strict, allowing it to recognize a wider variety of sequences with m6A modifications.^34^ Structurally, IGF2BP1 contains four K homology (KH) domains (KH1–4) and two RNA recognition motifs (RRM-1 and RRM-2), which is different from the structures of other m6A readers such as YTHDFs and YTHDCs (Fig. 2A).^35^^,^^36^ Previous studies have shown that the KH domains mainly facilitate the recognition and binding of reader proteins to the target RNAs. For instance, the KH1 and KH2 domains facilitate IGF2BP1 binding to the 3′-UTR of actin beta (ACTB),^37^ while the KH3 and KH4 domains can assemble into an anti-parallel pseudo-dimer conformation, which is crucial for binding of IGF2BP1 to MYC mRNA.^34^^,^^37^^,^^38^ Meanwhile, the RRM domains potentially contribute to the stabilization of IGF2BP-RNA complexes.^39^ As an important m6A reader protein, IGF2BP1 is involved in various aspects of RNA fate, including RNA stability, translation, and localization.^40^ Mechanically, IGF2BP1 is capable of recruiting its target RNAs to cytoplasmic messenger ribonucleoprotein particles that subsequently condense into processing bodies and stress granules, thereby stabilizing related RNAs and up-regulating target gene expression (Fig. 2B).^37^ Moreover, IGF2BP1 can promote the translation of mRNAs. IGF2BP1 facilitates the nuclear export of m6A-modified mRNAs to the cytoplasm, where they can be recognized by ribosomes and undergo translation.^41^^,^^42^ IGF2BP1 also contributes to the recruitment of ribosomes to mRNAs, thus enhancing their translation.^34^^,^^43^ In addition, IGF2BP1 can regulate the function of m6A-modified RNAs through interactions with other molecules. IGF2BP1 could interact with RNA-binding regulatory peptide (RBRP) encoded by long noncoding RNA (lncRNA) LINC00266-1, thereby increasing IGF2BP1's ability to identify and bind to m6A-modified c-Myc RNA. This subsequently enhances c-Myc RNA stability and expression level, and eventually, promotes tumorigenesis.^34^

Besides regulating mRNA stabilization, splicing, export, and translation efficiency, m6A readers such as the YTH domain protein family could accelerate targeted mRNA decay by selective binding to m6A-modified mRNAs. However, interestingly, unlike other m6A readers, IGF2BP1 cannot promote mRNA degradation; instead, it stabilizes its target mRNA.^44^^,^^45^ Accumulating evidence demonstrated that IGF2BP1, which is up-regulated in many human cancers and associated with poor prognosis and treatment resistance in patients with cancers,^42^^,^^46^ plays critical roles in various cancers including lung cancer,^47^ endometrial cancer,^48^ breast cancer,^49^ bladder cancer,^50^ and liver cancer.^51^ This abnormal expression of IGF2BP1 promotes the m6A modifications of its target mRNAs and thus increases their stability. This in turn leads to the unbalanced expression of oncogenes and tumor-suppressor genes, subsequently promoting the occurrence and development of tumors.^44^^,^^52^ Therefore, IGF2BP1 might be a promising target for anti-tumor therapy.

In this review, we summarize the current research regarding the biological roles of IGF2BP1 and the underlying molecular mechanisms by which IGF2BP1 regulates cancer hallmarks. In addition, we highlight the clinical significance of IGF2BP1 as a biomarker for tumor prognosis and a potential target for tumor therapy.

## Biological roles of IGF2BP1 in embryonic development

IGF2BP1 is highly expressed during embryogenesis, including that of mice, porcine, goat, and even humans.^53^, ^54^, ^55^, ^56^ During the development of mice embryos, IGF2BP1 expression peaks at embryonic day 12.5, and gradually decreases before it subsequently disappears in adults.^37^^,^^57^ Specifically, at embryonic day 12.5, IGF2BP1 is highly expressed in the muscle, epithelia, brain, and limb buds. Notably, IGF2BP1 expression was nearly abolished in the adult organism, although modestly expressed in the spleen, lung, and brain in 16-week-old mice.^37^^,^^58^ IGF2BP1 plays a crucial role in embryonic development by regulating mRNA stability and translation. Therefore, IGF2BP1 is specifically expressed in “oncofetal”, since IGF2BP1 has negligible expression in adult organs.^59^ Hansen et al found that *IGF2BP1* deficient mice had dwarfism and significantly reduced survival as well as impaired gut development, which subsequently leads to smaller body and perinatal death.^57^ Meanwhile, methyltransferase 16 (METTL16)/IGF2BP1 axis-mediated m6A methylation is essential for early embryonic development, as it controls the cell cycle progression of hematopoietic stem and progenitor cells.^55^ In embryo development studies using porcine, Y-box binding protein 1 (YBX1) could promote early embryo development owing to methyltransferase 3 (METTL3)/IGF2BP1 axis-mediated m6A methylation which decreases the mRNA expression level of maternal genes and increases those of zygotic genes.^54^ Moreover, recognition of m6A methylation by IGF2BP1 is essential for the development of porcine prenatal skeletal muscle. Similarly, IGF2BP1 also plays a crucial role in human development. Pillas et al have revealed that the SNP rs9674544 of IGF2BP1 is vital for primary tooth development in infancy.^60^ Meanwhile, IGF2BP1 induces a reversal of the hemoglobin of adults to a more fetal-like phenotype.^57^^,^^61^ Taken together, these studies have demonstrated that IGF2BP1 is significantly important in embryonic development.

## The role of IGF2BP1 in hallmarks of cancer

The occurrence and development of cancer are extremely complex processes closely related to the hallmarks of cancer, such as sustaining proliferative signaling, evading growth suppressors, resisting cell death, inducing angiogenesis, activating invasion and metastasis, avoiding immune destruction, deregulating cellular metabolism, and unlocking phenotypic plasticity.^62^^,^^63^ Extensive studies have revealed that m6A modification is involved in cancer hallmarks by regulating tumor-related gene expression at post-transcriptional stages.^64^, ^65^, ^66^ IGF2BP1, as a vital m6A reader, specifically recognizes and binds to m6A methylation mRNAs, leading to the increase of their stability and translation.^34^ IGF2BP1 is frequently overexpressed in various types of cancer and is associated with poor prognosis.^44^ Next, we will further summarize the specific roles of IGF2BP1 in different hallmarks of cancer (Table 1).

**Table 1:** The roles of m6A reader IGF2BP1 in cancer's hallmarks.

Cancer type	m6A sites	Targets	Mechanisms	Hallmarks	Reference
Pancreatic cancer	/	MYC	Stability	Sustaining proliferative signaling	67
Lung cancer	3′-UTR	SRF, MYC	Stability	Sustaining proliferative signaling	47,68
Non-small-cell lung cancer	/	TK1	Stability	Sustaining proliferative signaling	69
Renal cell carcinoma	Near stop codon	S1PR3	Stability	Sustaining proliferative signaling	70
Ovarian cancer	3′-UTR /	UBA6 circASXL1	Stability	Sustaining proliferative signaling	71,73
Hepatocellular carcinoma	3′-UTR /	LYPD1 PAQR4	Stability	Sustaining proliferative signaling	72,74
Esophageal squamous cell carcinoma	3′-UTR	MKK6 MAPK14	Stability	Sustaining proliferative signaling	41
Gastric cancer	/ 3′-UTR	MYC NDUFA4	Stability	Sustaining proliferative signaling	75,76
Colorectal cancer	/	MYC, FZD6	Stability	Sustaining proliferative signaling	77,78
Osteosarcoma	/	MCAM	Stability	Apoptosis resistance	79
Renal cell carcinoma	/	ZHX2	Stability	Apoptosis resistance	80
Hepatocellular carcinoma	/	circMAP3K4	Translation	Apoptosis resistance	81
Gastric cancer	/	ABL	Stability	Apoptosis resistance	82
Hepatoblastoma	3′-UTR	SLC7A11	Stability	Ferroptosis resistance	83
Breast cancer	/	GPX4	Stability	Ferroptosis resistance	84
Non-small-cell lung cancer	/	GPX4	Stability	Ferroptosis resistance	85
Ovarian cancer	3′-UTR /	NAT10 FTH1	Translation Stability	Ferroptosis resistance	86,87
Hepatocellular carcinoma	/	PARK7	Stability	Ferroptosis resistance	88
Bladder cancer	/	STAT3	Stability	Ferroptosis resistance	89
Gastric cancer	3′-UTR	NRF2	Stability	Ferroptosis resistance	90
Nasopharyngeal carcinoma	/	LINC00313	Stability	Ferroptosis resistance	91
Gastric cancer	/	lncRNA THAP7-AS1	Translation	Invasion and metastasis	92
Cervical cancer	/	SYVN1	Stability	Invasion and metastasis	93
Breast cancer	/	CPT1A, lncRNA MIR210HG	Stability	Invasion and metastasis	49,94
Esophageal squamous cell carcinoma	/	INHBA	Stability	Invasion and metastasis	95
Oral squamous cell carcinoma	3′-UTR	BMI1	Stability	Invasion and metastasis	43
Endometrial cancer	/	BDNF	Stability	Invasion and metastasis	96
Colorectal cancer	CDS	SOGA1	Stability	Deregulated metabolism	97
Osteosarcoma	3′-UTR	ERRα	Stability	Deregulated metabolism	98
Intrahepatic cholangiocarcinoma	/	NFAT5	Stability	Deregulated metabolism	99
Clear-cell renal cell carcinoma	3′-UTR	LDHA	Stability	Deregulated metabolism	100
Cervical squamous cell carcinoma	/	SIRT3	Stability	Deregulated metabolism	101
Gastric cancer Hepatocellular carcinoma	/ 3′-UTR	c-Myc	Stability	Deregulated metabolism	102,103
Colon cancer, gastric cancer, hepatocellular carcinoma, bladder cancer	3′-UTR	PD-L1	Stability	Immune evasion	104, 105, 106, 107
Colorectal cancer	/	AXIN2	Stability	Immune evasion	108
Non-small-cell lung cancer	3′-UTR	BUB1B	Stability	Immune evasion	109
Lung adenocarcinoma	/	ECE2	Stability	Immune evasion	110
Ovarian cancer	/	circNFIX	Stability	Immune evasion	111
Hepatocellular carcinoma	/	CD47 MIR4435-2HG	Stability	Unlocking phenotypic plasticity	112,113
Colorectal cancer	/	DDX27	Stability	Unlocking phenotypic plasticity	114
Hepatocellular carcinoma	/	MGAT5	Stability	Unlocking phenotypic plasticity	115
Non-small-cell lung cancer	/	BUB1B	Stability	Unlocking phenotypic plasticity	109
Breast cancer	/	c-Myc	Stability	Unlocking phenotypic plasticity	116
Gastric cancer	3′-UTR	IQGAP3	Stability	Unlocking phenotypic plasticity	117

## Sustained cell proliferation

Sustained cell proliferation is one of the most fundamental characteristics of tumor cells.^118^ The pro-mitotic growth signals, which are necessary for the entry of cells into the active proliferative state, are regulated strictly in normal cells, thereby keeping the cell number in balance. However, tumor cells can up-regulate these growth signals, subsequently inducing abnormal and uncontrolled cell proliferation.^119^^,^^120^ Aberrant expression of the m6A reader IGF2BP1 has been observed in various types of cancers, including gastric cancer (GC), lung cancer, non-small-cell lung cancer (NSCLC), and HCC, and contributes to sustained tumor cells' abnormal proliferation by regulating the expression of oncogenes including MYC, serum response factor (SRF), thymidine kinase 1 (TK1), ALKBH5, and LY6/PLAUR domain containing 1 (LYPD1).^47^^,^^68^^,^^69^^,^^72^^,^^75^^,^^121^

MYC is a vital oncogene and usually, its high expression in cancer is closely related to tumorigenesis.^122^ Recent studies have found that IGF2BP1 can enhance the expression of MYC, a vital oncogene highly expressed in cancers, by promoting m6A methylation-mediated mRNA stability, thereby accelerating tumor cell growth.^47^^,^^67^^,^^75^^,^^78^ This regulation has been found in various cancers, including lung cancer, GC, pancreatic cancer, and colorectal cancer (CRC).^47^^,^^67^^,^^75^^,^^78^ Through miRNome analysis, Simon et al revealed that IGF2BP1 could compete with miR-23a-3p and miR-125a-5p to bind to the 3′-UTR of serum response factor (SRF) mRNA, thereby blocking their function in triggering the decay of SRF mRNA. This leads to the stabilization of SRF mRNA, ultimately increasing lung cancer cell proliferation.^68^ In NSCLC, RCC, and ovarian cancer, m6A/IGF2BP1 mediated mRNA stabilization of thymidine kinase 1 (TK1), sphingosine-1-phosphate-receptor 3 (S1PR3), and ubiquitin-like modifier activating enzyme 6 (UBA6),^69^, ^70^, ^71^ while in HCC, ALKBH5-mediated m6A demethylation could suppress the IGF2BP1-mediated LYPD1 mRNA stabilization, thereby decreasing the proliferation potential of HCC cells.^72^

Mitogen-activated protein kinase (MAPK) and phosphatidylinositol 3-kinase/protein kinase B (PI3K/Akt) signaling pathways are crucial for sustained tumor cell proliferation, as they can promote cell cycle progression and angiogenesis while suppressing cell apoptosis.^123^^,^^124^ IGF2BP1 can regulate MAPK and PI3K/Akt signaling pathways, leading to an increase in cell proliferation. Zhang et al found that ribosomal protein S15 (RPS15) is a critical component of ribosomal biogenesis that plays a vital role in translation. RPS15 could bind to the KH domain of IGF2BP1 and subsequently, RPS15/IGF2BP1 complex enhances the translation of the p38/MAPK signaling pathways genes, including mitogen-activated protein kinase 14 (MAPK14) which can encode p38 protein, and MAP kinase kinase 6 (MKK6), by directly binding to their 3′-UTR region. This ultimately activates the p38/MAPK pathway, leading to enhanced proliferation and metastatic potentials of esophageal squamous cell carcinoma cells.^41^ In HCC, down-regulation of ALKBH5 decreases IGF2BP1-dependent m6A methylation of adipoQ receptor family member 4 (PAQR4) mRNA, thus increasing its mRNA stability and expression level. PAQR4 in turn promotes tumorigenesis by activating PI3K/AKT pathway.^74^

Moreover, IGF2BP1 can activate the cell cycle to sustain cell growth. For example, IGF2BP1 expression suppression causes G0/G1 cell cycle arrest in GC cells by decreasing NADH dehydrogenase (ubiquinone) 1 alpha subcomplex 4 (NDUFA4) mRNA stability, thereby inhibiting cell proliferation.^76^ Similarly, high expression of m6A methylation reader IGF2BP1 is also vital for promoting the expression levels of oncogenes such as MYC and fascin actin-bundling protein 1 (FSCN1).^50^^,^^125^^,^^126^ Intriguingly, the m6A sites of circular RNAs (circRNAs) and lncRNAs can also be recognized by IGF2BP1. For instance, METTL3/m6A/IGF2BP1 enhances the expression of circASXL1, a ceNRA, thereby sequestrating miR-320d from active Rac GTPase activating protein 1 (RACGAP1). This in turn enhances RACGAP1 expression and activates the RACGAP1/PI3K/Akt axis, and subsequently, promotes ovarian cancer cell proliferation and invasion potentials.^73^ Meanwhile, LINC00659 promotes tumor cell growth by regulating the stability of frizzled class receptor 6 (FZD6) mRNA in digestive tract tumors in an IGF2BP1-dependent manner.^77^ Hence, IGF2BP1 serves as a critical mediator for sustained tumor cell proliferation by regulating multiple growth signal pathways.

## Cell death resistance

Cell death resistance is one vital of fourteen hallmarks of cancer which is crucial for tumorigenesis as well as tumor resistance to chemotherapy.^127^^,^^128^ Cell death can be classified into accidental cell death, which is an uncontrolled process of cell death, and regulated cell death, which is an autonomous and controlled cell death.^129^^,^^130^ Regulated cell death, which includes apoptosis, necroptosis, autophagy, pyroptosis, and ferroptosis, plays a vital role in maintaining an organism's homeostasis by clearing damaged, stressed, malignant, and infected cells.^131^^,^^132^ The balance between cell growth and cell death, which is controlled by multiple controlled signaling pathways, is crucial for maintaining the size of the cell population.^133^ Since the correlation between m6A methylation and apoptosis was first elucidated, increasing numbers of researchers have focused on aberrant m6A modification as crucial regulators of regulated cell death.^134^

Apoptosis is a regulated cell death characterized by a series of biochemical events that result in cell changes, including blebbing, cell shrinkage, chromatin condensation, nuclear fragmentation, DNA fragmentation, and the formation of apoptotic bodies.^135^ Apoptosis is controlled by apoptotic-related genes including the caspase family, B-cell lymphoma-2 (Bcl-2) family, and genes involved in the protein tyrosine phosphatase (PTEN)/PI3K/AKT pathway as well as nuclear factor kappa B (NF-κB) pathway, and is one of the most extensively studied and understood regulated cell death.^136^^,^^137^ IGF2BP1 can bind to the coding region or 3′-UTR of beta-transducin repeat-containing protein 1 (βTrCP1) mRNA, enhancing its mRNA stability and expression level. This subsequently activates the Skp1-Cullin1-F-box protein (SCF)^βTrCP^ E3 ubiquitin ligase and promotes the degradation of the IκB protein to release NF-κB, ultimately inhibiting the apoptosis of CRC cells.^138^^,^^139^ METTL3/m6A/IGF2BP1-mediated stabilization of melanoma cell adhesion molecule (MCAM) and zinc-fingers and homeoboxes 2 (Zhx2) mRNA suppressed apoptosis of osteosarcoma cells and RCC cells.^79^^,^^80^ IGF2BP1 could also recognize the m6A methylation site in the circular RNA mitogen-activated protein kinase kinase kinase 4 (circMAP3K4) and enhance its translation into a 455 amino acid (aa) protein (circMAP3K4-455aa), which in turn suppressed the cleavage and nuclear distribution of apoptosis-inducing factor (AIF). This ultimately inhibits cisplatin-induced apoptosis in HCC.^81^ In GC, METTL3 stabilized apoptotic protease-activating factor 1 (APAF1)-binding lncRNA (ABL) in an IGF2BP1-m6A dependent manner. ABL could bind directly to APAF1 and block apoptosome assembly and caspase-3/9 activation by competitively preventing the interaction of cytochrome c with APAF1, leading to apoptosis inhibition.^82^ Moreover, IGF2BP1 also reduces cell apoptosis in a variety of tumors including NSCLC and intracranial aneurysms.^69^^,^^139^, ^140^, ^141^, ^142^

Emerging evidence has shown that abnormal IGF2BP1 expression level is strongly associated with ferroptosis, a unique type of programmed cell death induced by imbalanced iron ion and/or dysregulated redox homeostasis, and characterized by cytological changes such as ruptured outer mitochondrial membrane, mitochondria shrinkage, and decreased or vanished of mitochondria cristae.^143^, ^144^, ^145^ Imbalanced iron ion homeostasis leads to the accumulation of lipid peroxides; meanwhile, impaired *de novo* glutathione synthesis and glutathione peroxide 4 (GPX4) down-regulates cellular redox potential to reduce the lipid peroxides. Liu et al have revealed that METTL3/IGF2BP1/m6A can stabilize the mRNA of solute carrier family membrane 11 (SLC7A11). Given that SLC7A11 is the core enzyme of system X_c_−, which facilitates the exchange of intracellular cystine with extracellular glutamate, a component for making glutathione, this stabilization boosts ferroptosis resistance in hepatoblastoma.^83^ IGF2BP1 could also increase tumor cell ferroptosis resistance by enhancing GPX4 mRNA stability in breast cancer and NSCLC cells.^84^^,^^85^ Liu et al found that IGF2BP1 promotes the translation of N-acetyltransferase 10 (NAT10) mRNA in an m6A-dependent manner in ovarian cancer cells, which in turn mediates the N4-acetylcytidine modification of Acyl-CoA thioesterase 7 (ACOT7) mRNA, leading to the modulation of fatty acid metabolism and the suppression of ferroptosis.^86^ Furthermore, IGF2BP1 can promote ferroptosis resistance by stabilizing ferritin heavy chain 1 (FTH1), signal transducer and activator of transcription 3 (STAT3), and parkinsonism-associated deglycase (PARK7) mRNAs in HCC, bladder cancer, ovarian cancer, and GC, respectively.^87^, ^88^, ^89^, ^90^

Autophagy is a highly conserved system in eukaryotes. Autophagy degrades damaged, unfunctional proteins, macromolecules, and cytoplasmic organelles, and thus is essential for cell maintenance and survival.^146^^,^^147^ Autophagy plays a “double-edged sword” role in cancer, with both tumor-suppressive and tumor-promoting effects depending on the context. In tumor initiation, autophagy could suppress tumorigenesis by degrading damaged organelles and misfolded proteins, while in the stage of tumor development, autophagy can maintain tumor growth and survival through various ways, including nutrients and energy supply, as well as resistance to hypoxic and oxidative stress.^148^^,^^149^ METTL3/m6A/IGF2BP1 axis suppresses autophagy and enhances stemness by enhancing the stabilization and up-regulation of LINC00313 in nasopharyngeal carcinoma cells.^91^ Although evidence regarding the role of IGF2BP1 in tumor cell autophagy is still lacking, Tang et al have shown that IGF2BP1 could induce autophagy in several pathological conditions, including Alzheimer's disease and osteogenesis of bone marrow mesenchymal stem cells.^150^^,^^151^ Thus, although further investigations are necessary, these facts point out the possible involvement of IGF2BP1 in tumor cell autophagy.

Overall, while whether IGF2BP1 is also involved in the regulation of other types of cell death, such as necrosis, necroptosis, and pyroptosis, remains to be elucidated, studies have demonstrated the crucial roles of the m6A/IGF2BP1 axis in regulated cell death. Moreover, given that cell death resistance is a characteristic of tumor cells that contributes significantly to tumorigenesis, the negative regulation of IGF2BP1 on regulated cell death emphasizes the pivotal role of IGF2BP1 as an oncogene.

## Activation of invasion and metastasis

Invasion and metastasis are important traits of malignant transformation that lead to tumor cell dispersion.^152^ Metastasis is a multi-stage and complex process that includes the following steps: local invasion, intravasation, survival in circulation, extravasation, colonization, and metastatic outgrowth.^153^^,^^154^ METTL3/m6A/IGF2BP1-mediated stabilization of lncRNA THAP7-AS1 enhances the binding between cullin 4B (CUL4B) and importin β1, thereby promoting CUL4B protein nucleus localization, and subsequently, suppressing miR-320a and miR-22-3p expression levels. This ultimately activates the PI3K/AKT signaling pathway and promotes the invasion and metastasis potentials of GC cells.^92^ Meanwhile, Sui et al have deciphered that m6A/IGF2BP1-mediated mRNA stabilization of synoviolin 1 (SYVN1) could be suppressed by lncRNA LRRC75A-AS1. Down-regulation of SYVN1 leads to the accumulation of NLR family pyrin domain containing 3 (NLRP3) protein by suppressing its ubiquitination/proteasomal degradation, leading to the activation of NLRP3/IL-1β/Smad2/3 axis, and eventually, epithelial–mesenchymal transition in cervical cancer.^93^ Moreover, m6A modification is significantly enriched in carnitine palmitoyltransferase 1A (CPT1A) and lncMIR210HG transcripts. These modifications could be recognized by IGF2BP1, leading to the increase of their mRNA levels, and subsequently, metastasis of breast cancer cells.^49^^,^^94^ Furthermore, IGF2BP1 could promote inhibin subunit beta A (INHBA) expression level by stabilizing its mRNA, thereby triggering the activation of the INHBA/Smad2/3 signaling pathway, and eventually promoting the migration and invasion capacities of esophageal squamous cell carcinoma cells.^95^ Tian et al have shown that METTL3 promotes the stability of brain-derived neurotrophic factor (BDNF) mRNA via an m6A/IGF2BP1-mediated manner, followed by the activation of BDNF/TRKB signaling pathway, which is a positive regulator of epithelial–mesenchymal transition, thus increasing the metastatic potential of endometrial cancer cells.^96^ In oral squamous cell carcinoma, METTL3 increases IGF2BP1-dependent mRNA m6A methylation of B cell-specific Moloney murine leukemia virus integration site 1 (BMI1), a polycomb complex protein, thus promoting its mRNA translation by recruiting polysomes to BMI1 mRNA.^43^ In breast cancer as well as in head and neck squamous cell carcinoma, IGF2BP1 could also up-regulate invasion and metastasis potential by regulating metastatic-related protein (MRP) and Wnt/β-catenin pathways.^155^^,^^156^

Recent studies revealed that extracellular vesicles (EVs) could facilitate tumor metastasis by modulating the formation of pre-metastatic niches. In neuroblastoma and melanoma, IGF2BP1 promotes tumor metastasis by regulating the cargo of EVs.^157^ Specifically, IGF2BP1 could promote semaphorin 3A (SEMA3A) and serine hydroxymethyltransferase 2 (SHMT2) expression levels by stabilizing their mRNA, thereby regulating their protein levels in EVs. This can modulate the pro-metastatic microenvironment and promote metastasis in neuroblastoma cells.^157^ IGF2BP1 can selectively regulate certain RNAs, including activating transcription factor 2 (ATF2) and eukaryotic translation initiation factor 4A2 (EiF4a2) to enter EVs, thus affecting the composition of the RNA cargo in EVs. This subsequently accelerates the EV-induced metastasis process in melanoma cells.^158^ However, the role of IGF2BP1 as an m6A reader in promoting EVs-mediated metastasis has not been reported yet, and further investigation is warranted.

Collectively, IGF2BP1 could promote epithelial–mesenchymal transition as well as migration and invasion potentials of tumor cells by mediating m6A methylation; hence, the m6A/IGF2BP1 axis is beneficial for triggering tumor metastasis.

## Deregulated cellular energetics

Tumor cells often face severe tumor microenvironments, and thus potential to overcome stresses, such as hypoxia, oxidative stress, as well as deficiency in energy and nutrients is crucial to support their viability and rapid proliferation. To this end, tumor cells usually alter their metabolism.^159^^,^^160^ As first discovered by Warburg, normal cells mainly rely on glycolysis and following oxidative phosphorylation to produce energy; meanwhile, despite its inefficiency in producing energy, tumor cells prefer aerobic glycolysis and following lactate fermentation even in the presence of abundant oxygen.^161^^,^^162^ Furthermore, Warburg has also discovered that the glycolytic rate of tumor cells is 100 times higher than that of normal cells.^163^ Since then, studies have revealed that tumor cells not only alter their glucose metabolism but also their lipid as well as amino acid metabolism.^164^, ^165^, ^166^, ^167^ The m6A reader IGF2BP1 can trigger several tumor metabolic reprogramming. METTL16 and IGF2BP1 promote m6A modification of suppressor of glucose, autophagy associated 1 (SOGA1), remarkably increasing its stability and expression level. SOGA1 in turn promotes the expression of pyruvate dehydrogenase kinase 4 (PDK4), a key protein of glucose metabolism that is involved in the regulation of the pyruvate dehydrogenase complex (PDC), thus enhancing CRC cell's glycolysis.^97^ Moreover, IGF2BP1 suppresses estrogen-related receptor alpha (ERRα) mRNA decay by directly binding to its 3′-UTR in an m6A-dependent manner, subsequently enhancing oxygen consumption rate, ATP levels, glucose consumption, and lactate production of osteosarcoma cells, and thus contributes to osteosarcoma cell resistance to doxorubicin.^98^ Gao et al found that the METTL3/m6A/IGF2BP1 axis is crucial for stabilizing the mRNA of nuclear factor of activated T cells 5 (NFAT5), which can up-regulate the expression of the gluconeogenesis-related genes glucose transporter type 1 (GLUT1) and phosphoglycerate kinase 1 (PGK1), thereby promoting glycolytic reprogramming in intrahepatic cholangiocarcinoma.^99^ Studies using clear-cell RCC and GC showed that IGF2BP1 directly interacted with lactate dehydrogenase A (LDHA) and c-MYC mRNAs and enhanced their stability, thus accelerating aerobic glycolysis.^100^^,^^102^

IGF2BP1 is also involved in tumor cell lipid metabolic reprogramming. IGF2BP1 can increase the stability of sirtuin 3 (SIRT3) mRNA by recognizing its m6A site. This subsequently enhances the expression of SIRT3, a key enzyme in fatty acid synthesis that changes the confirmation and acetyl-binding ability of acetyl-coenzyme A-carboxylase 1 (ACC1) by recognizing its acetylated sites and promotes their deacetylation. This eventually leads to increased lipid metabolism in cervical squamous cell carcinoma.^101^

Moreover, IGF2BP1 is also closely correlated with glutamine metabolism. For example, tyrosine-protein phosphatase nonreceptor type 13 (PTPN13) binds to IGF2BP1 and reduces the intracellular concentration of IGF2BP1 which can directly bind to c-Myc mRNA, thereby decreasing c-Myc mRNA stability and expression level. PTPN13/IGF2BP1/c-Myc signaling pathway subsequently can enhance glutamine metabolism, resulting in increased glutathione levels and the glutathione/glutathione disulfide ratio in HCC cells.^103^

Recently, Elcheva et al found that IGF2BP1 plays a vital role in drug metabolic processes, as it could sensitize leukemia cells to the alkylating agents by regulating the expression levels of aldehyde dehydrogenase 1A1 (ALDH1A1), a factor that promotes tumor glycolysis, in a post-transcriptional regulation way.^168^^,^^169^ However, whether or not IGF2BP1 as an m6A reader is involved in the drug metabolic process remains unclear.

Taken together, IGF2BP1 plays a significant role in regulating metabolic reprogrammings in cancer cells as it could control glucose metabolism, lipid metabolism, and glutathione metabolism mediating m6A modification. Its role in these metabolic pathways makes IGF2BP1 a significant factor in tumor progression.

## Immune evasion

The immune system plays a critical role in the surveillance and elimination of tumor cells, a process known as immunosurveillance.^170^, ^171^, ^172^, ^173^ In normal conditions, the immune system can continuously monitor the states of cells and tissues; furthermore, by immune surveillance, most new tumor cells can be recognized and eliminated.^119^^,^^174^ However, solid tumors have managed to avoid surveillance in the different arms of the immune system and decrease immunological killing of cells, eventually evading eradication.^175^^,^^176^ Recently, the role of m6A methylation in tumor immune evasion has attracted great attention.^177^^,^^178^ METTL3/m6A/IGF2BP1 axis could enhance the mRNA stability and expression level of programmed cell death 1 ligand 1 (PD-L1) by promoting and recognizing m6A methylation in its 3′-UTR region. This subsequently deteriorates the immune escape and CD8^+^ T cell-mediated tumor cytotoxicity and apoptosis in colon cancer, GC, HCC, and bladder cancer.^104^, ^105^, ^106^, ^107^ Meanwhile, Zhai et al elucidated that IGF2BP1 promotes the mRNA stability of axis inhibitor 2 (AXIN2), a vital regulator of the Wnt/β-catenin signaling pathway, in an m6A-dependent way. Subsequently, the m6A/IGF2BP1 axis could enhance the expression of Dickkopf-related protein 1 (DKK1), a target of Wnt/β-catenin, to recruit myeloid-derived suppressor cells. The cells in turn significantly inhibit the function of T and natural killer cells to damage anti-tumor immunity in CRC.^108^ Furthermore, a study using HCC revealed that *IGF2BP1* knockdown can remarkedly increase the infiltration of immune cells, including CD8^+^ T cells, CD4^+^ T cells, F4/80^+^ macrophage, and CD56^+^ natural killer cells, thereby decreasing PD-L1 expression level and activating tumor immune microenvironment. Furthermore, a recent study demonstrated that IGF2BP1 could increase the stability of budding uninhibited by benzimidazoles 1 mitotic checkpoint serine/threonine kinase B (BUB1B) mRNA in an m6A-dependent way. IGF2BP1/BUB1B axis in turn enhances PD-L1 expression level and accelerates CD8^+^ T cell exhaustion, resulting in immunosuppression in NSCLC.^109^ In lung adenocarcinoma and ovarian cancer, m6A/IGF2BP1-mediated mRNA stabilizations of endothelin-converting enzyme 2 (ECE2) and circRNA nuclear factor I (circNFIX) are also crucial for tumor cells to enhance immunosuppression.^110^^,^^111^

## Unlocking phenotypic plasticity

Unlocking phenotypic plasticity refers to the ability of the organism to alter its phenotype in response to environmental changes. Unlocking phenotypic plasticity is a vital hallmark of cancer, contributing to tumor initiation, development, metastasis, and drug resistance.^179^^,^^180^ Tumor cells predominantly exhibit a proliferative phenotype when oxygen and nutrients are abundant in the early stage of tumor growth. As tumor cells proliferate and a hypoxic and nutrient-deficient environment emerges within, cells with plastic phenotypes transform into a more invasive mesenchymal phenotype, which allows tumor cells to break through the basement membrane and invade surrounding tissues and blood vessels.^181^ Fan et al found that METTL3 could enhance cluster of differentiation 47 (CD47) mRNA stability through IGF2BP-mediated m6A modification, thus giving rise to epithelial–mesenchymal transition and increased resistance to microwave ablation in HCC cells subjected to sublethal heat treatment.^113^ Furthermore, in CRC, cervical cancer, and endometrial cancer, m6A/IGF2BP1-mediated mRNA stabilizations of DEAD-box helicase 27 (DDX27), BDNF, and SYVN1 are also vital for tumor cells to enhance epithelial–mesenchymal transition.^93^^,^^96^^,^^114^

Tumor cell plasticity is closely intertwined with the maintenance of stem cell features. These undifferentiated or poorly differentiated cells divide incessantly and, to a certain extent, are not restricted by the normal cell differentiation program, thereby contributing to uncontrolled cell proliferation.^182^ IGF2BP1 is reported to be a critical regulator of stem cell features in various tumor types.^109^^,^^112^^,^^114^, ^115^, ^116^, ^117^^,^^168^ IGF2BP1 promotes the self-renewal and initiation of leukemia stem cells as well as the sensitivity of leukemia cells to the alkylating agents by regulating the expression of ALDH1A1, HOXB4, and MYB.^168^ Moreover, IGF2BP1 recognizes the m6A sites on lncRNA MIR4435-2HG and increases its expression. This subsequently promotes the interaction of MIR4435-2HG with nucleolar protein 58 (NOP58), a vital component of box C/D small nucleolar ribonucleoproteins, thereby protecting NOP58 from degradation. MIR4435-2HG/NOP58 axis subsequently enhances rRNA 2′-O-Me levels and the translation efficiency of MYC and insulin-like growth factor 1 receptor (IGF1R), ultimately contributing to the progression and stemness of HCC.^112^ Recent studies disclosed that IGF2BP1 could participate in CRC stemness maintenance. IGF2BP1 significantly increases the m6A-modified DDX27 mRNA stability, contributing to the stemness and metastasis of CRC.^114^ Moreover, IGF2BP1 can recognize and bind to the mRNA of alpha-1,6-mannosylglycoprotein 6-beta-N-acetylglucosaminyltransferase (MGAT5), BUB1B, c-Myc, and IQ motif containing GTPase-activating protein 3 (IQGAP3) through m6A modification, thereby maintaining the stemness of HCC, NSCLC, and breast cancer stems cells.^109^^,^^115^, ^116^, ^117^ Collectively, these studies suggest that IGF2BP1 regulates phenotypic plasticity and disrupts differentiation in tumor cells.

## Tumor microenvironment

The tumor microenvironment (TME) is a dynamic and complex ecosystem that includes tumor cells, endothelial cells, immune cells, fibroblasts, extracellular matrix, various cytokines, and chemokines.^183^ The dynamic interaction of these constituents promotes tumor cell growth, metastasis, and responses to anti-tumor therapies.^184^ Thus, TME is crucial in the initiation and progression of tumors, as well as in their response to therapy. Studies have found that IGF2BP1 can alter the TME by stabilizing tumor-related mRNAs.^185^ In the hypoxic TME, hypoxia enhances IGF2BP1 expression levels by regulating the ability of hypoxia-inducible factor-1alpha (HIF-1α to bind to the *IGF2BP1* promoter, subsequently increasing the proliferation and invasion potentials of melanoma cells, while decreasing their apoptotic rates.^186^ Zhu et al found that IGF2BP1 could inhibit c-Myc mRNA decay by directly binding to its 3′-UTR in an m6A-dependent manner, thereby maintaining the stemness of breast cancer stem cells in a hypoxic microenvironment.^116^ Moreover, IGF2BP1 promotes aerobic glycolysis and following lactate fermentation to decrease the pH value of the TME. This acidic TME can hinder the function of immune cells and immunosuppressive cells, including CD8^+^ T cells, myeloid-derived suppressor cells, and regulatory T cells, thereby promoting immune evasion. Meanwhile, circFAM13B competitively interacts with the KH3-4 regions of IGF2BP1, leading to decreasing the binding of IGF2BP1 with the 3′-UTR of pyruvate kinase muscle isozyme M2 (PKM2), a crucial enzyme in glycolysis, in an m6A-dependent manner. This in turn destabilizes PKM2 mRNA stability and suppresses glycolysis-induced acidic TME.^187^ Furthermore, IGF2BP1 can promote the progression of CRC, osteosarcoma, intrahepatic cholangiocarcinoma, and clear-cell RCC by stabilizing the mRNAs of factors that enhance glucose metabolism, including those of SOGA1, ERRa, NFAT5, and LDHA.^97^, ^98^, ^99^, ^100^ In summary, IGF2BP1-mediated m6A modification plays a multifaceted role in the TME, acting as a key driver in tumorigenesis and cancer progression.

## IGF2BP1 is a potential biomarker in human cancer

As mentioned above, IGF2BP1 usually functions as an oncogene in human cancers and relates to different aspects of cancer hallmarks. Furthermore, up-regulated IGF2BP1 expression is frequently found in a broad range of cancers, and is associated with poor prognosis. Previous studies reported that m6A regulators, including IGF2BP1, show a correlation with poor prognosis and advanced clinic grade in ovarian cancer, NSCLC, and GC.^188^, ^189^, ^190^, ^191^, ^192^, ^193^ IGF2BP1 is also significantly elevated in malignant melanomas, and its high expression is strongly correlated with metastasis and poor prognosis.^194^^,^^195^ Kim et al revealed that IGF2BP1 expression level was positively associated with various malignant tumor behaviors, including a higher tumor stage, a shorter overall survival time, and a higher risk of recurrence in cutaneous squamous cell carcinoma.^196^ Meanwhile, using 266 clinical CRC specimens, Chen et al found that high IGF2BP1 expression was correlated with therapeutic resistance, a higher clinic stage, and a shorter overall survival.^197^ Moreover, a positive correlation of IGF2BP1 expression with a higher risk score, advanced tumor grade, shorter overall survival, and poor prognosis has also been found in uterine corpus endometrial carcinoma.^198^ Up-regulation of IGF2BP1 is also closely related to poor overall survival and high tumor grades in RCC and osteosarcomas,^199^^,^^200^ as well as with short disease-free survival and overall survival in intrahepatic cholangiocarcinoma.^201^^,^^202^ Similarly, analysis of IGF2BP1 expression in clinical patients confirmed that elevated IGF2BP1 expression could enhance lymph node metastasis and recurrence and accompany poor prognosis in lung adenocarcinoma and HCC patients.^203^^,^^204^ Thus, IGF2BP1 has attracted attention for its potential to serve as a biomarker for diagnosis and prognosis for abovementioned cancers, as well as for other cancers including uveal melanoma, breast cancer, neuroblastoma, esophageal cancer, and cervical cancer.^49^^,^^205^, ^206^, ^207^, ^208^, ^209^ The correlation of IGF2BP1 with human cancers, as well as their clinical characteristics, are summarized in Table 2. Together, IGF2BP1 is significantly up-regulated in human cancers and is associated with tumor progression and poor prognosis. These suggest the potential of applying IGF2BP1 as a marker for tumor diagnosis and prognosis prediction.

**Table 2:** IGF2BP1 expression and clinical characteristics in human cancers.

Cancer type	Expression	Clinical characteristics	Reference
Ovarian cancer	Up-regulated	Overall survival, relapse-free survival, and clinic stage	188, 189, 190
Non-small-cell lung cancer	Up-regulated	Overall survival and disease-free survival	191,192
Gastric cancer	Up-regulated	Advanced clinic stage	193
Melanoma	Up-regulated	Survival and tumor grade	194,195
Cutaneous squamous cell carcinoma	Up-regulated	Tumor grade, overall survival, and recurrence	196
Colorectal cancer	Up-regulated	Therapeutic resistance, tumor grade, and overall survival	197
Uterine corpus endometrial carcinoma	Up-regulated	Risk score, tumor grade, and tumor grade	198
Renal cell carcinoma	Up-regulated	Overall survival and tumor grade	199
Osteosarcoma	Up-regulated	Overall survival and tumor grade	200
Intrahepatic cholangiocarcinoma	Up-regulated	Overall survival and disease-free survival	201
Testicular germ cell tumor	Up-regulated	Overall survival and progression-free survival	202
Lung adenocarcinoma	Up-regulated	Lymph node metastasis and recurrence	203
Hepatocellular carcinoma	Up-regulated	Lymph node metastasis and recurrence	204,210
Uveal melanoma	Up-regulated	Overall survival and disease-free survival	209
Breast cancer	Up-regulated	Survival	49
Neuroblastoma	Up-regulated	Survival and tumor grade	206
Esophageal cancer	Up-regulated	Tumor-node-metastasis and survival	207
Cervical cancer	Up-regulated	Overall survival	208

## IGF2BP1 is a potential target of anti-tumor therapy

Resistance to anti-tumor therapy, including immunotherapy, radiotherapy, targeted therapy, and chemotherapy, is the main cause of anti-tumor treatment failure and is correlated with poor prognosis.^211^^,^^212^ Therefore, it is particularly important to improve the sensitivity of anti-tumor treatment. IGF2BP1 is significantly up-regulated in various tumor cells and has attracted attention as a potential target gene for anti-tumor therapy. IGF2BP1 could promote leukemia cell resistance to doxorubicin, cytarabine, and cyclophosphamide, and enhance melanoma cell resistance to dacarbazine, temozolomide, vinblastine, and etoposide^168^^,^^213^ Furthermore, IGF2BP1 desensitized BRAF^V600E^ melanoma cells to vemurafenib, BRAF-inhibitors, and BRAF-MEK inhibitors, and CRC cells to 5-FU, irinotecan (CPT-11), and oxaliplatin.^214^^,^^215^ Besides in an m6A-unrelated pathway, recent studies showed that IGF2BP1 could confer resistance to chemotherapy and radiotherapy by regulating the mRNA stability through its function as an m6A reader, thereby controlling the expression level of target genes associated with chemotherapy and radiotherapy resistance.^51^^,^^185^^,^^216^^,^^217^ Moreover, it is also negatively correlated with the sensitivity of other anti-tumor therapeutic strategies, such as immunotherapy and hyperthermia.^218^^,^^219^ The correlation between the IGF2BP1/m6A axis and tumor therapeutic resistance, along with the signaling pathways involved, was summarized in Table 3.

**Table 3:** Roles of IGF2BP1 in therapeutic strategies of cancer.

Cancer type	Expression	Target	Resistance	Reference
Ovarian cancer	Up-regulated	METTL3/IGF2BP1/BIRC5 IGF2BP1/IGF2/MEK/ERK	Cisplatin	216,232
Oral squamous cell carcinoma	Up-regulated	PI3K/Akt/mTOR	Cisplatin	220
Seminoma	Up-regulated	METTL3/IGF2BP1/TFAP2C	Cisplatin	221
Neuroblastoma	Up-regulated	IGF2BP1/genes related to proliferation	Cisplatin, topotecan	222
Liver cancer	Up-regulated	PRTM3/IGF2BP1/HEG1	Oxaliplatin	51
Rhabdomyosarcoma	Up-regulated	IGF2BP1/cIAP1	TNFα	223
Osteosarcomas	Up-regulated	IGF2BP1/ERRα	Doxorubicin	98
Pancreatic cancer	Up-regulated	IGF2BP1/SH3BP5-AS1/CTBP1	Gemcitabine	224
Nasopharyngeal carcinoma	Up-regulated	IGF2BP1/AKT2	Taxol	225
Ovarian cancer	Up-regulated	Let-7/IGF2BP1/MDR1	Taxanes	226
Colorectal cancer	Up-regulated	IGF2BP1/LDHA	Hyperthermia	219

IGF2BP1 could attenuate the tumor suppressive effect of cisplatin and induce cisplatin resistance in ovarian cancer, oral squamous cell carcinoma, seminoma, and endometrial cancer cells by regulating its target mRNA stabilization. Specifically, METTL3 post-translationally stabilizes the mRNA of the baculoviral inhibitor of the apoptosis protein repeat-containing 5 (BIRC5), a member of the inhibitor of the apoptosis protein (IAP) family, in an m6A/IGF2BP1-mediated manner. Subsequently, high expression of BIRC5 can increase cisplatin-chemoresistance cells by reducing tumor cell apoptosis.^216^ In oral squamous cell carcinoma and seminoma cells, m6A/IGF2BP1-mediated mRNA stabilization of estrogen-related receptor alpha (ERRα) and transcription factor-activating enhancer-binding protein 2C (TFAP2C) can facilitate tumor cell survival upon cisplatin treatment.^220^^,^^221^ Similarly, IGF2BP1 can increase the mRNA stability and expression levels of various target genes related to cell proliferation by specifically binding to their mRNAs, thereby promoting neuroblastoma cell proliferation and inducing chemotherapeutic agent resistance, including cisplatin, cyclophosphamide, doxorubicin, etoposide, and topotecan.^222^ Meanwhile, IGF2BP1 could increase the stability of heart development protein with EGF-like domains 1 (HEG1) mRNA in an m6A-dependent way, which subsequently increases liver cancer cell resistance to oxaliplatin.^51^ Moreover, IGF2BP1 binds to the 5′-UTR internal ribosome entry site (IRES) region of cellular inhibitor of apoptosis 1 (cIAP1), a member of the inhibitor of apoptosis protein family, and enhances its translation. Subsequently, high expression of cIAP1 interferes with the rhabdomyosarcomas (RMS) cell apoptosis process, thereby leading to the resistance of RMS cells to tumor necrosis factor alpha (TNFα).^223^ IGF2BP1 mediates the mRNA stabilization of ERRα in an m6A-dependent way in osteosarcoma, thus increasing its expression level. The increased expression level of ERRα further results in metabolic reprogramming of osteosarcoma cells, such as the increase of the ATP levels, oxygen consumption rate, glucose consumption, and lactate generation. These metabolic changes enable tumor cells to survive better under the treatment of doxorubicin, reducing its killing effect, and thus inducing doxorubicin resistance of osteosarcoma cells.^98^ The down-regulation of IGF2BP1 also can render cancer cells resistant to gemcitabine, taxol, and taxanes in pancreatic cancer, nasopharyngeal carcinoma, and ovarian cancer.^224^, ^225^, ^226^ Zhang et al found that IGF2BP1 could bind to the m6A site on the 3′-UTR of the mRNA of lactate dehydrogenase-A (LDHA), a glycolysis rate-limiting enzyme, and enhance its stability. Subsequently, the IGF2BP1/LDHA axis can enhance glucose metabolism, thereby allowing tumor cells to better withstand hyperthermia, diminishing the cytotoxic impact of hyperthermia, and consequently leading to the development of hyperthermia resistance in CRC cells.^219^

Importantly, recent studies have found several small inhibitors targeting IGF2BP1 that show promising potential for anti-tumor treatment, including 2-[(5-bromo-2-thienyl) methyleneamino benzamide (BTYNB), (4-((1H-tetrazol-1-yl)methyl)phenyl) (3-(benzo[d][1,3]dioxol-5-ylamino)piperidin-1-yl)methanone (compound 7773), (4-(Benzo[d] [1,3]dioxol-5-yl)piperazin-1-yl) (4-((3-(hydroxymethyl)-1H-indol-1-yl) methyl)phenyl)methanone (AVJ16), and 2-[(8-bromo-5-methyl-5 H-[1,2,4]triazino[5,6-b]indol-3-yl)thio]-N-(1-phenylethyl)acetamide; C_20_H_18_BrN_5_OS; ChemBridge ID: 6896009).^227^, ^228^, ^229^ BTYNB could decrease the proliferation of leukemia cells while promoting their differentiation.^227^ Meanwhile, compounds 7773 and C_20_H_18_BrN_5_OS could suppress cell growth and migration potentials by obstructing IGF2BP1-dependent post-transcriptional stabilization of MYC mRNA in NSCLC, ovarian clear cell carcinoma, and ovarian cancer.^230^^,^^231^ Moreover, AVJ16, one of compound 7773 derivatives, can directly bind to a hydrophobic region at the protein's KH34 di-domain interface between the KH3 and KH4 domains of IGF2BP1. This interaction specifically, blocks the binding between IGF2BP1 and its target RNAs, such as Kirsten rat sarcoma viral oncogene homologue (Kras), ultimately suppressing tumorigenic properties of tumor cells. This finding suggests that a specific IGF2BP1 inhibitor is a potential anti-tumor therapeutic agent for IGF2BP1-expressing cancers.^229^ Collectively, these studies suggest that IGF2BP1 inhibitors can inhibit the growth and development of tumors, and thus targeting IGF2BP1 is a potential anti-tumor therapeutic strategy.

Despite its potential as an anti-tumor drug reported in several studies using preclinical models, there is no report regarding the use of IGF2BP1 inhibitor in clinical and/or clinical trials currently. Thus, further systematical pre-clinical and clinical studies are necessary for assessing the use of IGF2BP1 at the clinical level. However, clinical research on drugs targeting IGF2BP1 is still in its early stages, thus additional studies are needed to explore and select appropriate drug candidates targeting IGF2BP1. Furthermore, efforts are needed to further clarify more in-depth and specific molecular mechanisms of IGF2BP1 in various tumors, including its binding mode with target mRNAs, its regulation of downstream signaling pathways, and its dynamic changes during tumor progression. This will help conduct reliable preclinical studies and clinical trials.

## Conclusions and perspectives

**Figure 3:**
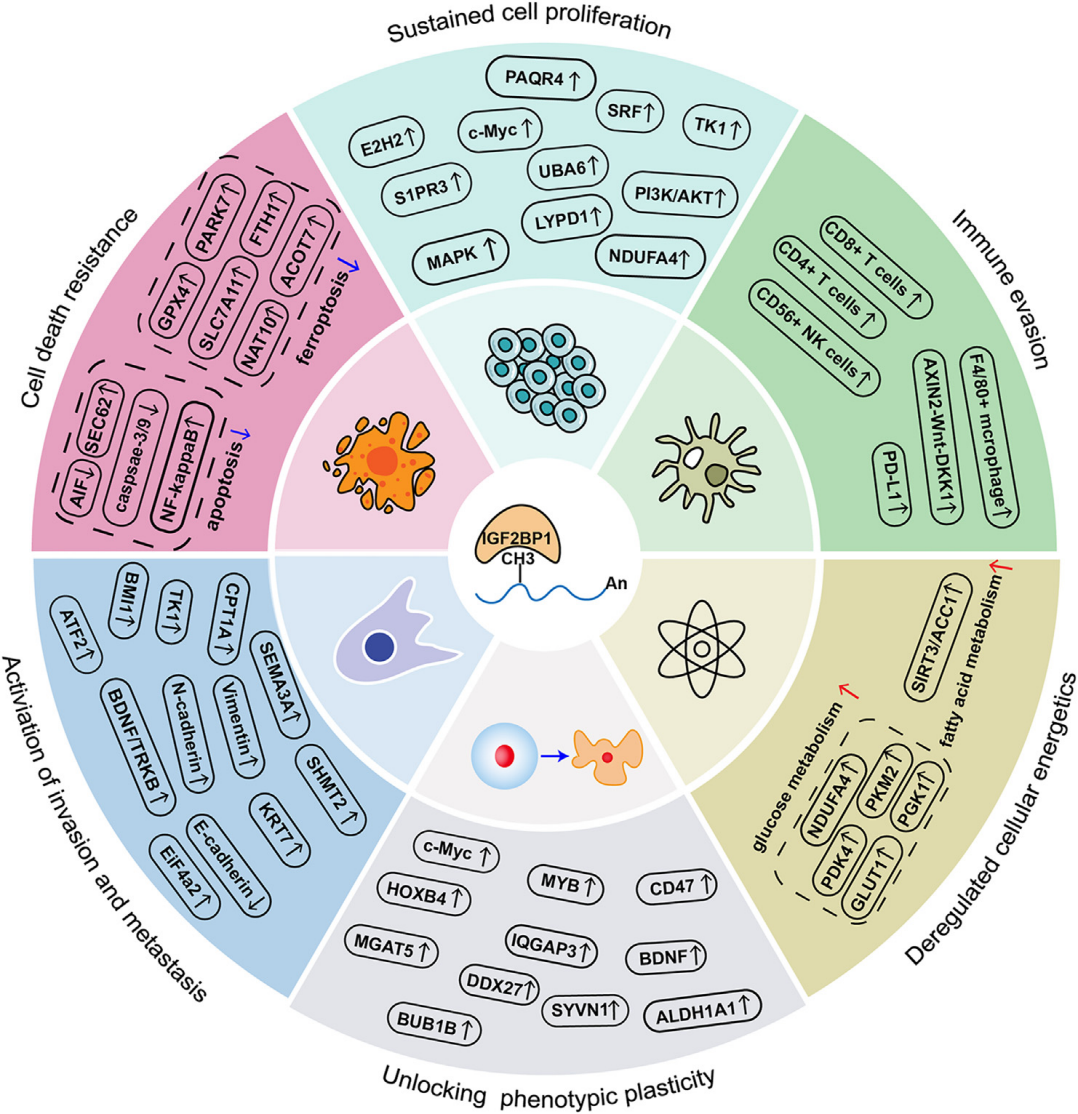
IGF2BP1's regulation on cancer's hallmarks. IGF2BP1 mainly regulates six cancer hallmarks, including sustained cell proliferation, cell death resistance, activation of invasion and metastasis, deregulated cellular energetics, immune evasion, and unlocking phenotypic plasticity.

With the rapid development of high-throughput sequencing technology, the molecular characteristics and functional mechanisms of m6A modification at the transcriptomic level are gradually revealed.^233^ Recently, m6A methylation modification has become a focus of cancer research, however, the role of IGF2BP1 in human cancers is not fully understood. As a vital m6A reader, IGF2BP1 can regulate the mRNA stability and translation of target genes by recognizing m6A-methylated sites, subsequently mediating their expression.^234^^,^^235^ As summarized above, IGF2BP1 is highly expressed in various cancers and is involved in the regulation of several hallmarks of cancer, including sustained cell proliferation, cell death resistance, activation of invasion and metastasis, deregulated cellular energetics, immune evasion, and unlocking phenotypic plasticity (Fig. 3).^69^^,^^74^^,^^95^^,^^98^^,^^106^^,^^134^ However, further studies are necessary to clarify the roles of the m6A/IGF2BP1 axis in other cancer hallmarks, such as angiogenesis, genome instability, mutation, polymorphic microbiomes, non-mutational epigenetic reprogramming, and enabling replicative immortality. Hence, further investigations are needed to study the role of m6A/IGF2BP1 in these hallmarks.

Accumulating studies demonstrate that high expression of IGF2BP1 is related to poor survival, high tumor grades, metastasis, and recurrence. Hence, IGF2BP1 is a potential biomarker for the diagnosis and prognosis prediction of cancer patients.^236^ Furthermore, IGF2BP1 is also a potential target for anti-tumor therapeutic strategy, as suppressing IGF2BP1 expression could significantly enhance tumor cell sensitivity to chemotherapeutic drugs, immunotherapy, and radiotherapy.^187^^,^^237^, ^238^, ^239^ However, the clinical translation of IGF2BP1-targeted therapy still faces many challenges and further in-depth studies are needed to verify the specificity, effectiveness, and safety of IGF2BP1-targeted therapy.

Collectively, despite further studies on the molecular mechanisms of IGF2BP1 need to be explored, the extensive functions of IGF2BP1 have demonstrated its significance in maintaining various cancer hallmarks, thus emphasizing its potential as prognostic markers and therapeutic targets.

## CRediT authorship contribution statement

**Li Qiu:** Writing – review & editing, Writing – original draft, Validation, Software, Methodology, Investigation, Conceptualization. **Shourong Wu:** Writing – review & editing, Writing – original draft, Validation, Supervision, Resources, Funding acquisition. **Lei Zhang:** Writing – original draft, Software, Methodology. **Wenfang Li:** Writing – review & editing, Validation, Software. **Debing Xiang:** Writing – review & editing, Writing – original draft, Supervision, Resources, Funding acquisition. **Vivi Kasim:** Writing – review & editing, Writing – original draft, Validation, Supervision, Resources, Funding acquisition, Conceptualization.

## Funding

This work was supported by grants from the National Natural Science Foundation of China (No. 82173029, 32270778, and 82372655), the Natural Science Foundation of Chongqing, China (No. CSTB2022NSCQ-MSX0611, CSTB2022NSCQ-MSX0612), and the Talent Project of Chongqing University Jiangjin Hospital (Chongqing, China) (No. 2024LJXM005).

## Conflict of interests

The authors declared no conflict of interests.
